# Fate of methyl methacrylate in rats.

**DOI:** 10.1038/bjc.1977.161

**Published:** 1977-07

**Authors:** H. Bratt, D. E. Hathway

## Abstract

Up to 88% of a single dose of methyl[14C]methacrylate in rats is expired as 14CO2 in 10 days (65% in 2 h), irrespective of the route of administration and of the specific labelling of the propylene residue of the molecule. The implications of this observation, and of the excretion of small amounts of [14C]methylmalonate, [14C]-succinate and probably of [14C]beta-hydroxyisobutyrate and 2-formylpropionate, and of the formation of [14C] normal, physiological metabolites that may be accounted for by anabolism both from 14CO2 and from [14C]acetate emergent from the citric acid cycle, are that the metabolic pathway concerned involves intermediary metabolism and relates to mitochondrial function. Present findings are discussed in relation to the imputations of a report of carcinogenic risk.


Br. J. Cancer (1977) 36, 114

### FATE OF METHYL METHACRYLATE IN RATS

H. BRATT AND D. E. HATHWAY

*From the Imperial Chemical Industries, Central Toxicology Laboratory, Alderley Park, Cheshire SK10 4TJ*

Received 7 February 1977 Accepted 7 March 1977

**Summary.—Up to 88% of a single dose of methyl[$^{14}$C]methacrylate in rats is expired as $^{14}CO_2$ in 10 days (65% in 2 h), irrespective of the route of administration and of the specific labelling of the propylene residue of the molecule. The implications of this observation, and of the excretion of small amounts of [$^{14}$C]methylmalonate, [$^{14}$C]-succinate and probably of [$^{14}$C]$\beta$-hydroxyisobutyrate and 2-formylpropionate, and of the formation of [$^{14}$C] normal, physiological metabolites that may be accounted for by anabolism both from $^{14}CO_2$ and from [$^{14}$C]acetate emergent from the citric acid cycle, are that the metabolic pathway concerned involves intermediary metabolism and relates to mitochondrial function. Present findings are discussed in relation to the imputations of a report of carcinogenic risk.**

METHYL METHACRYLATE and its homologous esters seem to be quite innocuous substances. Thus, Deichmann (1941) and Spealman *et al.* (1945) found that, in small laboratory animals, these substances were less acutely toxic than ethyl acetate with an s.c. $LD_{50}$ of 5000 mg/kg in rats, and that the most characteristic effects, caused by prolonged exposure to methyl methacrylate vapour, were degenerative changes of the liver. Chronic long-term toxicity testing of methyl methacrylate in dogs and rats by oral route (Borzelleca *et al.*, 1964) failed to reveal an increased mortality amongst the animals, or any histological changes. The dogs were given gelatin capsules containing methyl methacrylate, in solution in corn oil, for a period of 2 years, in amounts equivalent to 10, 100 and 1000–1500 parts/$10^6$ in the diet, and the rats received concentrations of 6–7, 60–70 and 2000 parts/$10^6$ in their drinking water.

However, the present, widespread usage of methyl methacrylate polymer for acrylic sheet and moulding material, and a report (Singh, Lawrence and Autian, 1972) that its i.p. administration to female rats on Days 5, 10 and 15 of gestation caused haemangiomas in some of the pups, make a better understanding of the biological fate of this monomer in mammals desirable.

### MATERIALS AND METHODS

*Chemicals.*—Methacrylic acid and succinic acid reference compounds were obtained from Koch-light Laboratories Limited, and were of Analar grade.

All reagents and solvents were of Analar grade or of the next highest quality available.

Methylmalonic acid, m.p. 135°C, was prepared by alkylation of diethyl malonate. Formyl-2-propionic acid (methylmalonic semialdehyde) was prepared by the method of Kupiecki and Coon (1960) and the corresponding alcohol, $\beta$-hydroxyisobutyric acid, was derived by $KBH_4$ reduction.

Methyl[1,3 - $^{14}$C]propylene - 2 - carboxylate with sp. act. 0·398 mCi/mmol, and with a chemical and radiochemical purity exceeding 98%, was synthesized from [1,3-$^{14}$C]acetone *via* the cyanohydrin, and methyl[2-$^{14}$C]-propylene-2-carboxylate with sp. act. 0·355 mCi/mmol, and with a chemical and radiochemical purity exceeding 98%, was synthesized similarly from [2-$^{14}$C]acetone, by our colleague Mr D. Greenslade of Imperial Chemical Industries Limited, Petrochemicals Division, Billingham, Cleveland. On account of the risk of polymerization, both radioactive chemicals were supplied in spectroscopic-grade ethanol solution, stabilized with $< 50$ parts/$10^6$ of hydroquinone. These solutions were stored at $-20$°C.

*Experiments in animals.*—Adult male rats (about 2 months old, 200 g body wt) were used (Alderley Park strain (Wistar-derived), specific-pathogen-free), and kept on a standard pellet diet.

(a) For the purpose of establishing the excretion-retention pattern of $^{14}C$ over a 10-day period, 3 rats were each administered intragastrically a 5·7-mg/kg body weight dose containing 4 $\mu$Ci of methyl [1,3-$^{14}C$]propylene-2-carboxylate in corn-oil solution. The animals were kept singly in glass metabolism cages (Jencons of Hemel Hempstead, Herts.). Unrestricted food and water were supplied, and the urine and faeces were collected separately in the dark, and frozen at $-20$°C. The exhaled air was drawn through a gas train, comprising successively two Dreschel bottles, each of which contained 75 ml of ethanol at $-70$°C to remove unchanged methyl[$^{14}C$]-methacrylate, and two $CO_2$ absorber Nilox columns (Jencons of Hemel Hempstead) each containing 500 ml of 2N NaOH. Products of excretion were collected for 10 days.

(b) Another 3 rats were each injected in a femoral vein with a 5·7 mg/kg body weight dose containing 4 $\mu$Ci of methyl[1,3-$^{14}C$]-propylene-2-carboxylate in ethanol solution (50 $\mu$l), and the excretion-retention pattern of $^{14}C$ was investigated over a similar period.

(c) Three animals were each injected in a femoral vein with a 6·8-mg/kg body weight dose containing 4 $\mu$Ci of methyl[2-$^{14}C$]-propylene-2-carboxylate in ethanol solution (50 $\mu$l), and the excretion-retention pattern of $^{14}C$ was studied over a 10-day period.

(d) Two rats were each given a single intragastric dose of methyl [2-$^{14}C$]propylene-2-carboxylate (120 mg/kg; 84 $\mu$Ci) as a corn-oil solution, and the excretion-retention pattern of $^{14}C$ was again studied over a 10-day period. More than 90% of the urinary $^{14}C$ was excreted during 48 h, and this urine was used for [$^{14}C$]metabolite identification.

*Measurement of radioactivity.*—An automated and computerized Intertechnique Model SL30 Liquid Scintillation Spectrometer was used for measurement of $^{14}C$, making use of standard channels-ratio quench-correction curves. Liquid samples were admixed with standard scintillator and radio-assayed direct, and samples of faeces were burnt in an Intertechnique "Oxymat", solid-sample oxidizer.

*Characterization of unchanged methyl methacrylate in the expired air.*—Ethanol from the cold traps was slowly evaporated under $N_2 < 15$°C, and samples of the concentrate were examined with a Pye model 104 gas chromatograph that was equipped with flame-ionization detection and coupled to an E.S.I. Nuclear 504 Radiogas detector. The column effluent was split in the ratio of 9 : 1 between the Radiogas detector and the flame-ionization detector. This gas chromatograph was fitted with glass columns (1·5 m long $\times$ 4 mm internal diameter), which were packed with 10% (w/w) of squalene on Chromosorb P (80–100 mesh size) and which were run at 75°C. All the columns were operated at a 30 ml/min flow-rate of a (95 : 5, v/v) Air–$CO_2$ mixture. Under these operating conditions, methyl methacrylate has a retention time of 5·8 min.

*Systematic separation of the urinary metabolites into fractions of chemically similar substances.*—A 50-ml sample of the combined urine from the 2 rats [(d) above] was evaporated to dryness under reduced pressure, at $< 35$°C. A solution of the residue in 5 ml of 0·1N KOH was percolated (20 drops/min) through a column (bed volume, 100 ml) of Amberlite IRA410 anion-exchange resin (14–52 mesh size, 1·40 mg-equiv/ml) in the $CH_3CO_2^-$ cycle, and the column was then washed (60 ml/h) with 250 ml of de-ionized water. The total eluate, which contained 54% of the urinary $^{14}C$, was retained. 500 ml of 3N acetic acid, when percolated through the column, stripped the remaining (46%) urinary $^{14}C$ from the anion-exchange resin.

*Thin-layer chromatography.*—A methanol solution of the evaporate from the bulked aqueous washings was spotted on $SiO_2$-gel GF thin-layer plates, which were run with butan-1-ol : acetic acid : water (4 : 1 : 1, v/v). A major [$^{14}C$] spot with an $R_F$ value of 0·27 was identified by chromatography and co-chromatography with reference material, as [$^{14}C$] urea and gave a characteristic yellow colour with Ehrlich's reagent. (Identification was confirmed by reverse isotope-dilution analysis of [$^{14}C$]urea nitrate in an aliquot portion of the methanol solution of the evaporate from the bulked aqueous washings). A minor [$^{14}C$] spot with an $R_F$ value of 0·14 on the developed plate was unidentified, but the presence of $H^{14}CO_3^-$ was established in further aliquot portions of the acidified urine in Conway-diffusion units.

Radioactivity in the 3N acetic acid fraction was not retained by percolation through a column (bed-volume, 50 ml) of Dowex 50W-X8 cation-exchange resin in the $H^+$ cycle. Accordingly, a methanol solution of the evaporate from the bulked effluent was spotted on $SiO_2$-gel GF thin-layer plates, which were run with the same solvent system. Chromatographic characteristics of the resulting [$^{14}C$] acidic metabolites of methyl [$^{14}C$]-methacrylate are exemplified in Table II. Radioactivity corresponding to each metabolite was measured in the eluate from the separate spots.

*Gas chromatography*.—Gas chromatography of the methyl esters of the foregoing [$^{14}C$] acidic metabolites was undertaken on the previously described gas chromatograph that was equipped with flame-ionization detection and coupled to an E.S.I. Nuclear 504 Radiogas detector. In this case, the gas chromatograph was fitted with glass columns (2 m long × 4 mm internal diameter), which were packed with 6% (w/w) of OV-101 on Supelcoport (80–100 mesh size) and which were run at 100°C. All the columns were operated at a 30 ml/min flow rate of a (95 : 5, v/v) Air–$CO_2$ mixture. Retention times of the principal methyl esters are shown in Table II.

*Analysis of triglyceride fat and cholesterol esters*.—A sample of lipid was saponified with alcoholic KOH (1 ml of 33% aq. KOH + 9 ml of ethanol/g of lipid), under $N_2$ in the dark in a sealed vessel, on a water-bath at 55°C for 30 min, when the cooled solution was extracted with ether to remove cholesterol and glycerol. The soaps were converted into free fatty acids by acidification, and the acid solution was re-extracted with *n*-hexane. The two solvent extracts were evaporated under reduced pressure, and the residues were dissolved in small volumes of *n*-hexane. Extracts were spotted separately on $SiO_2$-gel GF thin-layer plates, which were run either with *n*-hexane : diethyl ether : glacial acetic acid (90 : 20 : 1, v/v) or with chloroform : acetone : 5N $NH_3$ (10 : 80 : 10, v/v).

### RESULTS AND DISCUSSION

A high proportion of a dose of methyl methacrylate is fully oxidized in rats, and in an early investigation (Deichmann, 1941) no urinary metabolites were found. Thus, 84–88% of a single dose (5·7 mg kg) of methyl[1,3-$^{14}C$]propylene-2-carboxylate was expired from the lungs as $^{14}CO_2$ in 10 days, whether by parenteral or enteral administration (Table I), and up to 65% as $^{14}CO_2$ in 2 h. About half the remainder of the dose was excreted in the urine and the rest was retained by the body tissues at 240 h. Pulmonary excretion of unchanged methyl methacrylate accounted for less than 1·0% of the dose. The same pattern of excretion was observed when animals were dosed another labelled form of methyl methacrylate, *viz.* methyl[2-$^{14}C$]propylene-2-carboxylate (Table I). Within the limitations of the experiment, the rate of the initial phase of pulmonary excretion of $^{14}CO_2$ after an i.v. injection of methyl[1,3-

TABLE I.—*Excretion and Retention of Radioactivity in Rats after Administration of Methyl*[$^{14}C$]*methacrylate*

| | | | Recovery of $^{14}C$ (% of dose)* | | | | | |
|---|---|---|---|---|---|---|---|---|
| | | | | | Exhaled gases | | | |
| Form of $^{14}C$ label | Route of administration | Dose (mg/kg) | Urine | Faeces | $^{14}CO_2$ | Unchanged methyl[$^{14}C$] methacrylate | Carcass plus skin | Total |
| Methyl[1,3-$^{14}C$]-propylene-2-carboxylate | By stomach tube[a] | 5·7 | 4·7 | 2·7 | 88·0 | 0·1 | 4·1 | 99·6 |
| | i.v.[a] | 5·7 | 6·6 | 1·7 | 84·0 | 0·7 | 6·6 | 99·6 |
| Methyl[2-$^{14}C$]-propylene-2-carboxylate | i.v.[a] | 6·8 | 7·2 | 1·8 | 84·1 | 1·0 | 6·6 | 100·7 |
| | By stomach tube | 120·0 | 6·0 | 3·0 | 76·4 | 1·4 | [b] | — |

* 10 days after administration.
[a] The data displayed are representative of that obtained in several animals.
[b] Unmeasured.

FIG. 1.—Cumulative plots for the initial phase of pulmonary excretion of $^{14}CO_2$ in representative rats that had been dosed different radioactively labelled forms of methyl methacrylate. Each animal was given a single injection of either—methyl[1,3-$^{14}$C]propylene-2-carboxylate (5·7 mg/kg) ● or methyl[2-$^{14}$C]propylene-2-carboxylate (6·8 mg/kg) □ in a femoral vein at zero time (for details, see Methods section).

$^{14}$C]propylene-2-carboxylate did not differ significantly from that after i.v. injection of methyl[2-$^{14}$C]propylene-2-carboxylate (Fig. 1) and half the dose is expired as $^{14}CO_2$ in 90 min. It is reasonable to suppose that on the basis of this experimental evidence, all three propylene C atoms of methacrylate were being metabolized *in vivo* by the same sequence of biotransformations. Moreover, the pattern of excretion is not altered by a much larger intragastric dose (120 mg kg) of methyl[2-$^{14}$C]methacrylate (Table I) but in this case the compound was metabolized more slowly, and pulmonary excretion of unchanged methyl methacrylate accounted for 1·4% of the dose, compared with 0·1% for the smaller dose (5·7 mg/kg).

Hence there is a strong supposition that the biological fate of methyl[$^{14}$C]methacrylate in mammals implicates the pathways and cycles of intermediary metabolism. The scheme that is suggested for methacrylate degradation in Fig. 2 represents the preferred pathway, in which the branched-chain methylmalonyl CoA, which is known to be formed in valine catabolism, is converted by methylmalonyl mutase into succinyl CoA. Hence, by that means all 4 C atoms belonging to methacrylate would enter the citric acid cycle simultaneously and be oxidized into $CO_2$, but some anabolism of acetate into normal, physiological metabolites might be expected.

The fact that *in vivo* the ready esterification of methacrylate by CoA directs the entry of oxygen to the carbon atom, which is in $\beta$-position to the carboxyl function, makes less likely the possibility of $\alpha$-hydroxylation, which applies to the case of acrylonitrile, and of $\alpha,\beta$-dihydroxylation, which has been proposed (Pantůček, 1969) for methacrylate degradation. Whilst an $\alpha,\beta$-dihydroxy compound is formed in the biosynthesis of valine from pyruvate, the next reaction step in this case is dehydration, and not the C-deformylation suggested in Pantůček's (1969) scheme for methacrylate. In fact, Pantůček's (1969) arsenite-inhibited *in vitro* preparation may be somewhat unrealistic, and addition of small amounts of fumarate and malate to that preparation does not stimulate the citric acid cycle in

FIG. 2.—Scheme for the degradation of methacrylate in mammals.

the Krebs sense. Had the scheme leading to pyruvate production applied to [$^{14}$C]-methacrylate degradation in rats, then small amounts of [$^{14}$C]lactic acid might have been expected to have been excreted in the urine, but this was not the case.

The rest of the evidence is consistent with the scheme (Fig. 2) for the degradation of methacrylate *in vivo*. Thus, after administration of methyl[2-$^{14}$C]propylene-2-carboxylate, that fraction of the urine which comprises the [$^{14}$C]acidic metabolites (Table II) was found to contain, in addition to [$^{14}$C]methacrylic acid (0·8% of the dose), 1·4% of [$^{14}$C]methylmalonic acid ([$^{14}$C]isosuccinic acid), which arises by hydrolysis of methylmalonyl CoA (Fig. 2), 0·2% of [$^{14}$C]succinic acid from the hydrolysis of succinyl CoA (Fig. 2), and two other minor constituents, which occupy the same part of the chromatogram (Table II) as β-hydroxyisobutyric acid and formyl-2-propionic acid (methylmalonic semialdehyde) (Fig. 2). The $^{14}CO_2$ would appear to be the source of substantial amounts (> 2·0% of the dose) of [$^{14}$C]urea in the neutral fraction of the urine, as well as of a small quantity of $H^{14}CO_3^-$. At 10 days, $^{14}$C is retained in the body only by the adipose and liver tissues, and in those biological situations $^{14}$C is associated both with the corresponding hydrolysis products and with the unsaponifiables. This evidence suggests that, in such an experi-

TABLE II.—*Thin-layer Chromatographic Properties of Major Urinary Acidic Metabolites of Methacrylate, the Gas-chromatographic Characteristics of their Methyl Esters, and their Relative Proportions*

| Acidic metabolites | $R_F$ values on $SiO_2$-gel plates developed with butan-1-ol:acetic acid:water (4 : 1 : 1, v/v) | GC-retention times of methyl esters (min) Column coated with OV-101 and run at 100°C | Relative proportions of [$^{14}$C]metabolites (% of dose) |
|---|---|---|---|
| Methacrylic acid | *0·10 | 2·0 | 0·8 |
| Unidentified acid | 0·21 | | 0·4 |
| β-Hydroxyisobutyric acid | 0·39 | | 0·2 |
| Methylmalonic acid (isosuccinic acid) | 0·43 | 5·1 | 1·4 |
| Methylmalonic semialdehyde | 0·50 | | 0·1 |
| Succinic acid | 0·53 | 7·4 | 0·2 |

* Characteristically shaped, diffuse spot.

ment, some of the [$^{14}C$]acetate generated has been elaborated into triglyceride fat and cholesterol esters, and in fact materials consistent with [$^{14}C$] free fatty acids have been detected in alkaline hydrolysates of depot fat.

Thus, the rapid oxidation of [$^{14}C$]-methacrylate into $^{14}CO_2$, which has been encountered in the present investigation, seems to be consistent with the suggested scheme (Fig. 2). That the generation of $^{14}CO_2$ was incomplete is due to body compartmentalization, whereby a small amount of pulmonary excretion of the unchanged ester occurred, four [$^{14}C$]metabolic intermediates were excreted in the urine, and normal, physiological metabolites were anabolized both from $^{14}CO_2$ and from [$^{14}C$]acetate emerging from the citric acid cycle. On the assumption that the metabolic route (Fig. 2) does in fact apply in the way which has been described, it follows that exogenous methacrylate from the large amounts of administered methyl methacrylate is metabolized by the body in exactly the same way as the small amounts of endogenous methacrylate that are formed in the course of valine catabolism. This pathway is concerned with intermediary metabolism and relates to mitochondrial function.

Hence present evidence implies, but does not prove, that it is rather unlikely that methyl methacrylate metabolism would yield damaging reactive metabolites, since an acceptable pathway has been proposed which is independent of the microsomal and cytoplasmic enzymes that are usually concerned with foreign compound metabolism. The report of Singh *et al.* (1972) is accordingly difficult to reconcile and, in practice, i.p. injection would never be selected as the route for the testing of embryonal–foetal toxicity and teratogenicity *per se* (Hathway, 1975), since a compound under examination might be absorbed from the peritoneum into the foetuses direct, and not *via* the placenta.

We wish to express our gratitude to the other sponsors, including Altulor and Ugilor (France), Degussa, Resart Ihm A.-G. and Rohm G.m.b.H. (Germany), Montedison (Italy), Dupont de Nemours and Rohn & Haas (U.S.A.), and Sumitomo (Japan).

### REFERENCES

BORZELLECA, J. F., LARSON, P. S., HENNIGAR, G. R., CRAWFORD, E. M. & SMITH, E. B. (1964) Studies on the Chronic Toxicity of Monomeric Ethyl Acrylate and Methyl Methacrylate. *Toxicol. appl. Pharmacol.*, **6**, 29.

DEICHMANN, W. (1941) Toxicity of Methyl, Ethyl and *n*-Butyl Methacrylate. *J. ind. Hyg. Toxicol.*, **23**, 343.

HATHWAY, D. E. (1975) Embryonal and Neonatal Pharmacology. In *Foreign Compound Metabolism in Mammals*. Ed. D. E. Hathway. London: Chemical Society, 3, 658.

KUPIECKI, F. P. & COON, M. J. (1960) Methylmalonic Semialdehyde. *Biochem. Prep.*, **7**, 69.

PANTŮČEK, M. (1969) On the Metabolic Pathway of Methyl Methacrylate. *FEBS Lett.*, **2**, 206.

SINGH, A. R., LAWRENCE, W. H. & AUTIAN, J. (1972) Embryonic–foetal Toxicity and Teratogenic Effects of a Group of Methacrylate Esters in Rats. *J. Dent. Res.*, **51**, 1632.

SPEALMAN, C. R., MAIN, R. J., HAAG, H. B. & LARSON, P. S. (1945) Monomeric Methyl Methacrylate Studies on Toxicity. *Ind. Med.*, **14**, 292.


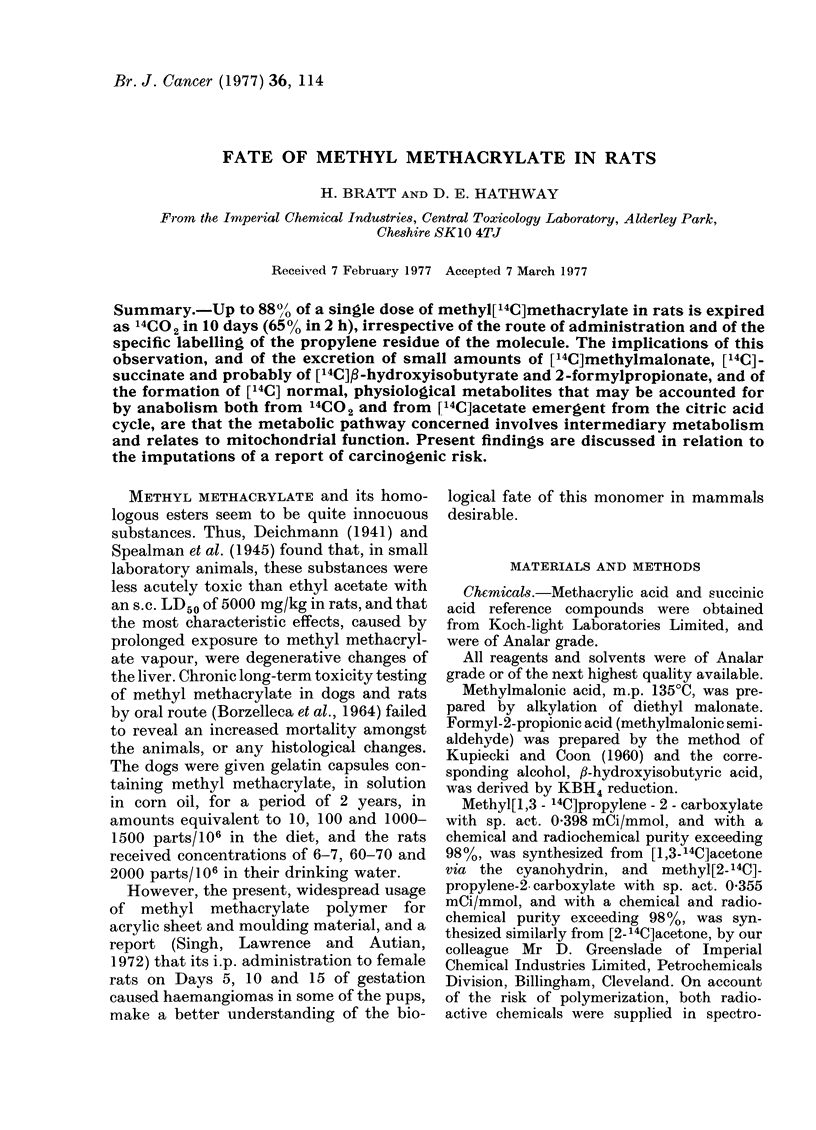

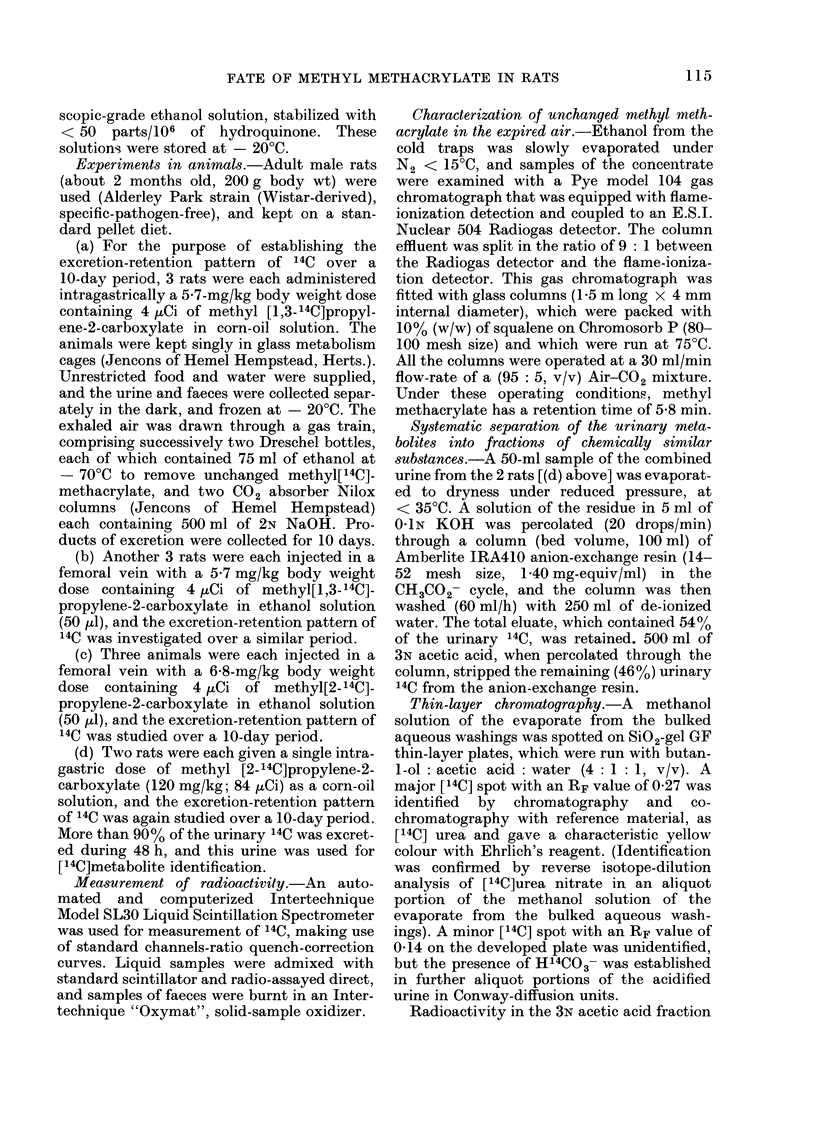

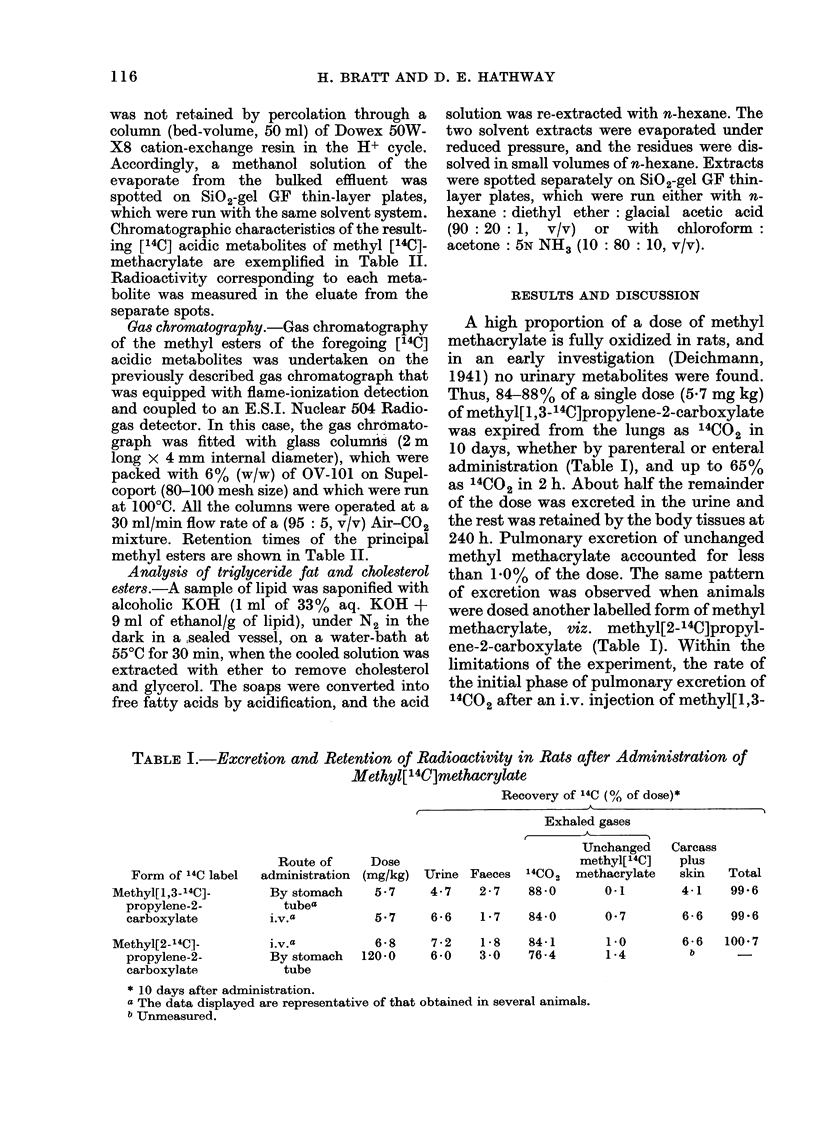

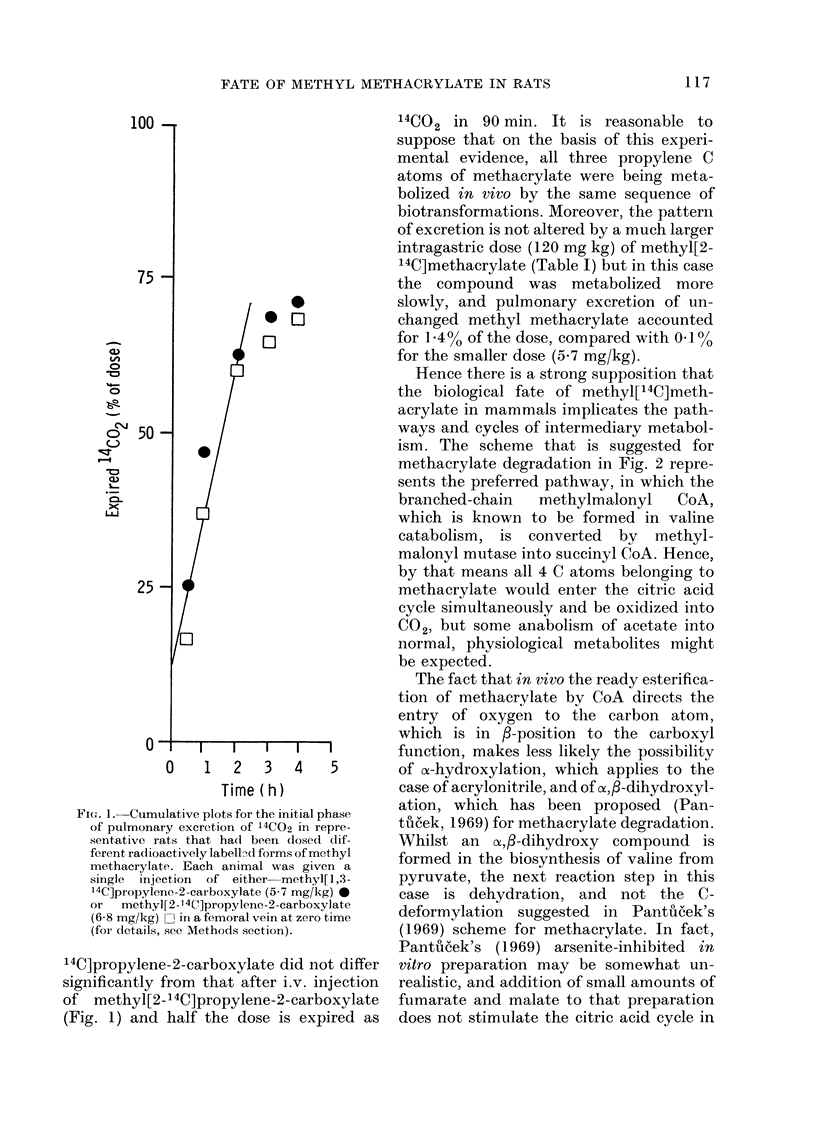

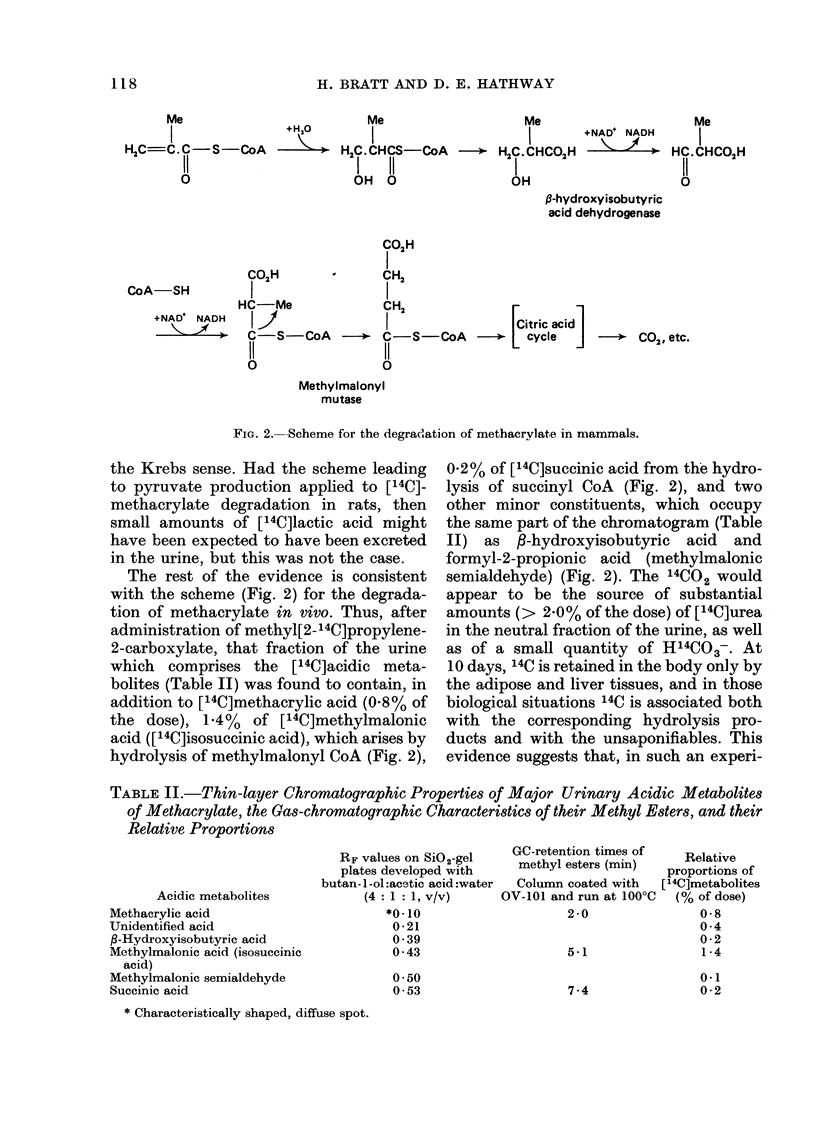

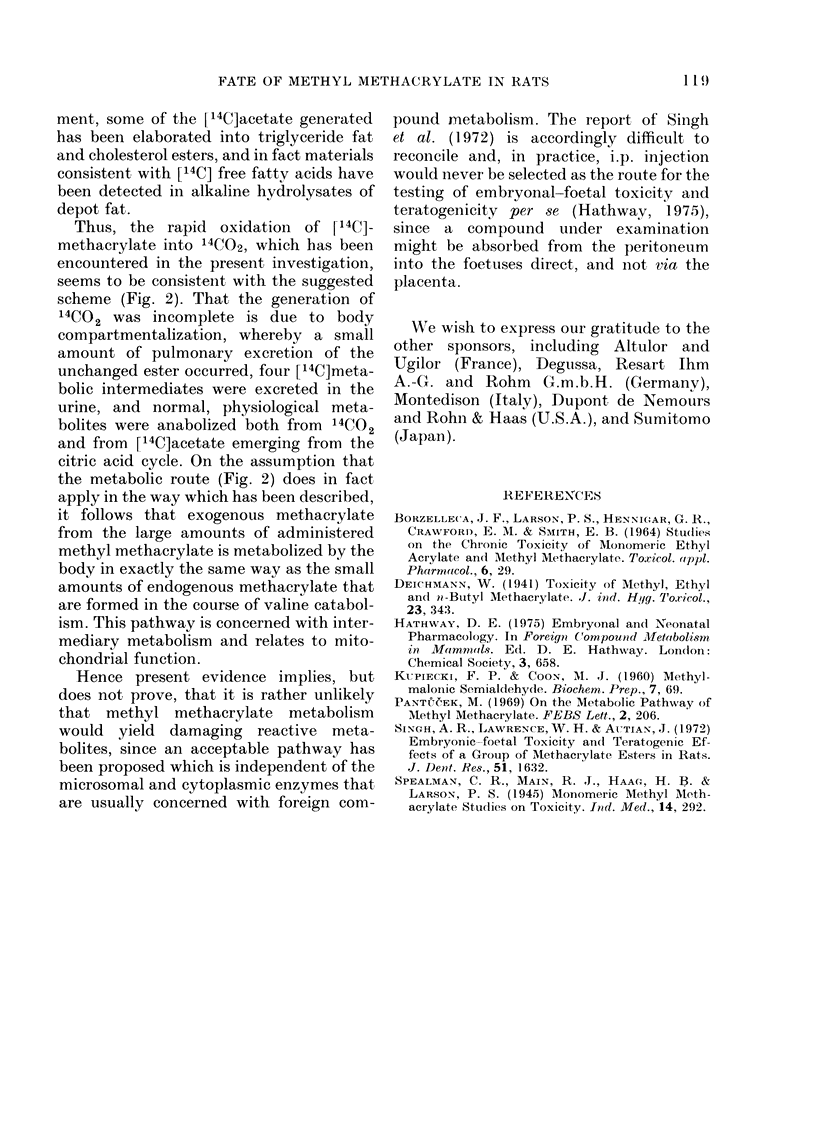


## References

[OCR_00663] Pantůcek M. (1969). On the metabolic pathway of methylmethacrylate.. FEBS Lett.

[OCR_00667] Singh A. R., Lawrence W. H., Autian J. (1972). Embryonic-fetal toxicity and teratogenic effects of a group of methacrylate esters in rats.. J Dent Res.

